# *Clostridium tertium* Peritonitis and Concurrent Bacteremia in a Patient With a History of Alcoholic Cirrhosis

**DOI:** 10.1177/2324709617731457

**Published:** 2017-09-14

**Authors:** S. Scott Sutton, Mark Jumper, Ansal Shah, Babatunde Edun

**Affiliations:** 1 University of South Carolina, Columbia, SC, USA; 2 Dorn VA Medical Center, Columbia, SC, USA

**Keywords:** Clostridium tertium, peritonitis, alcoholic cirrhosis

## Abstract

Spontaneous bacterial peritonitis (SBP) is a recognized cause of morbidity and mortality in cirrhotic patients. Enterobacteriaceae have been isolated from the majority of peritonitis cases and the gram negative aerobe *Escherichia coli* is the most commonly isolated organism. Anaerobic organisms are rarely isolated because of the high oxygen tension in ascetic fluid. We report a patient with a history of alcoholic cirrhosis who developed SBP and concurrent bacteremia with the anaerobe *Clostridium tertium*. The patient was successfully treated with intravenous antibiotics and was discharged home on oral ciprofloxacin. This case report is unique in that it is the fourth documented *Clostridium tertium* SBP case, utilized MALDI-TOF mass spectrometry for organism identification, and susceptibility testing for select antibiotics was performed.

## Introduction

Primary spontaneous bacterial peritonitis (SBP) is a recognized cause of morbidity and mortality in cirrhotic patients. SBP is an acute bacterial infection of the ascites fluid and is known to affect patients with cirrhosis from any cause, including alcoholic cirrhosis.^1,2^ The pathophysiology of bacterial introduction in the ascitic fluid is not known; however, cirrhosis predisposes the development of gastrointestinal bacterial overgrowth and increased intestinal permeability.^3,4^ Enterobacteriaceae have been isolated from the majority of peritonitis cases, and the gram-negative aerobe *Escherichia coli* is the most commonly isolated organism.^3,4^ While Enterobacteriaceae are commonly encountered, gram-positive organisms such as streptocococi and enterococci are sometimes found. Anaerobic organisms are rare because of the high oxygen tension in ascetic fluid.^3-5^ However, there are reports of anaerobic SBP infections. We report the case of a patient with a history of alcoholic cirrhosis who developed SBP and concurrent bacteremia with the anaerobe *Clostridium tertium*.

## Case Report

A 60-year-old man was admitted with worsening abdominal pain, nausea, vomiting, fever, and grossly extended abdomen accompanied by 1+ bilateral lower extremity edema. His symptoms had been ongoing for 1 week. Contributory medical history was significant for alcoholic cirrhosis and umbilical hernia surgery approximately 2 months prior to current admission. Temperature on admission was 102.2°F, otherwise his vitals were stable with no signs of sepsis or hemodynamic instability. Abdominal exam revealed diffuse tenderness and positive fluid thrill. His only subjective complaint was pain. Laboratory findings on admission are listed in Table 1, with abnormalities including serum white blood cell count and albumin. The patient’s cirrhosis severity was rated as a Child-Pugh Class B. Initial paracentesis fluid study results are listed in Table 2. The patient’s urinalysis was negative and nondiagnostic. Empiric antibiotic therapy was initiated with intravenous (IV) cefotaxime. The antibiotics were subsequently changed to IV vancomycin and meropenem due to persistent fever and abdominal pain. A repeat ultrasound-guided paracentesis was conducted on day 4 of admission, with 2 liters of fluid removed. Ascitic fluid cultures grew out anaerobic gram-positive rods. On day 5, the organism was identified as *C tertium* by matrix-assisted laser desorption/ionization-time of flight (MALDI-TOF) mass spectrometry. Following isolation of the anaerobic organism, ciprofloxacin and metronidazole were added to the vancomycin pending susceptibility testing and meropenem was discontinued. Over the next 24 hours, the patient improved clinically, with defervescence and leukocytosis resolution. Repeat cultures on day 7 were negative. Susceptibility testing was conducted using E-test, which showed that the *C tertium* was sensitive to vancomycin, ciprofloxacin, trimethoprim/sulfamethoxazole, meropenem, and clindamycin. IV antibiotics were continued until repeat cultures were finalized as negative, and the patient was discharged on oral ciprofloxacin.

**Table 1.** Laboratory Results Upon Admission.

Parameters	Readings	Normal Ranges
White cell count	15.2	3.6-11.1 K/mm^3^
Hemoglobin	13.9	12.9-16.1 g/dL
Platelet	196	165-353 K/mm^3^
Sodium	137	135-145 mmol/L
Potassium	3.5	3.5-5.1 mmol/L
Blood urea nitrogen	22	7-26 mg/dL
Creatinine	1.1	0.5-1.3 mg/dL
Calcium	9.0	8.4-10.2 mg/dL
Albumin	2.7	3.5-5.0 g/dL
Total bilirubin	5.6	0.2-1.2 mg/dL
Alkaline phosphatase	105	40-150 U/L
Alanine aminotransferase	44	0-55 U/L
Aspartate aminotransferase	74	5-34 U/L
International normalized ratio	1.4	0.8-1.2

**Table 2.** Paracentesis Fluid Studies.

Parameters	Readings
Total cells	23 777 cells/mm^3^
Nucleated cells	15 707 cells/mm^3^
Polymorphonuclear leukocytes	84%
Absolute polymorphonuclear leukocytes	13 193 cells/mm^3^
Lymphocytes	9%
Red blood cells	8064 cells/mm^3^
Total protein	1.6 g/dL

## Discussion

*Clostridium tertium* is a ubiquitous gram-positive bacillus isolated in soil and the gastrointestinal tract. *C tertium* distinguishes itself among the clostridia as a non–toxin-producing, aerotolerant species.^5^ Typically, *C tertium* does not have pathogenic potential, and its role as a human pathogen is uncertain.^6,7^ Infection with the organism has been rare after initial description in 1917. However, *C tertium* has documented cases of SBP, intraabdominal infection, enterocolitis, meningitis, septic arthritis, pneumonia, and necrotizing fasciitis.^5-13^ Case reports have identified neutropenia, mucosal injury, β-lactam antibiotics (third-generation cephalosporins), cytotoxic chemotherapy, and severe liver disease as predisposing factors for *C tertium* infection.^5^ While scare, case reports document that *C tertium* is a cause of SBP. Our case report of a patient with alcoholic cirrhosis adds to the literature regarding *C tertium* SBP. Previously published case reports include the following:

- Butler and Pitt reported a case of *C tertium* SBP in a 42-year-old female with a history of cirrhosis.^8^ The patient was admitted for management of hepatic encephalopathy with subsequent development of peritonitis. Clinical and microbiological cure was achieved with the cephamycin antibiotic cefoxitin.
- Miller et al reported 32 cases of *C tertium* bacteremia over a 7-year span.^5^ All 32 patients presented with fever, 59% had one or more abdominal symptoms, 9 had diarrhea (including 5 with a positive test for *Clostridium difficile* toxin), 9 had abdominal pain, 5 had nausea, and 1 had constipation. Twenty-nine of the 32 cases involved neutropenic patients, all of which were status post chemotherapy within 9 to 21 days before the onset of *C tertium* bacteremia. The 3 nonneutropenic patients had underlying host factors disposing them to *C tertium* infection: (1) chronic alcoholism and end-stage liver disease, (2) systemic lupus erythematosus receiving high-dose corticosteroids and recent placement of a percutaneous gastrostomy tube, and (3) Crohn’s disease. Four patients died within 1 week after the isolation of *C tertium*, including the patient with SBP. The patient with SBP was a 43-year-old patient with end-stage liver disease with concurrent bacteremia.
- Victor and Opal reported 43 patients with SBP during a 5-year period.^10^ Alcoholic liver disease was the underlying cause in 72% of the cases, and Enterobacteriaceae accounted for 66% of the cases. There were 2 cases of anaerobic infections (one patient had *C tertium* SBP with concurrent bacteremia). The overall mortality rate was 65% (mortality rates were not discussed regarding individual cases).

**Figure 1.**
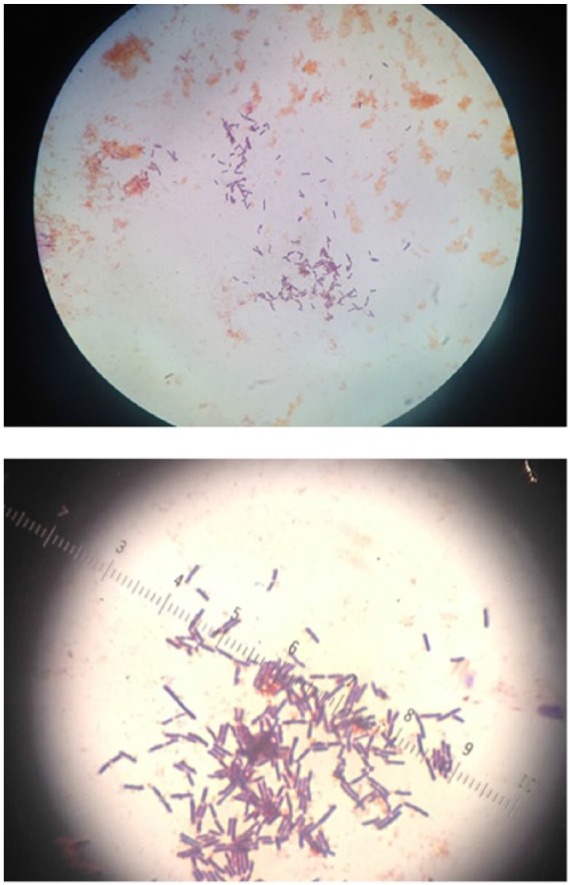
Gram stain of *Clostridium tertium*, which is an anaerobic, motile, gram-positive bacterium. It is easily decolorized in gram-stained smears and can be mistaken for a gram-negative organism.

**Figure 2.**
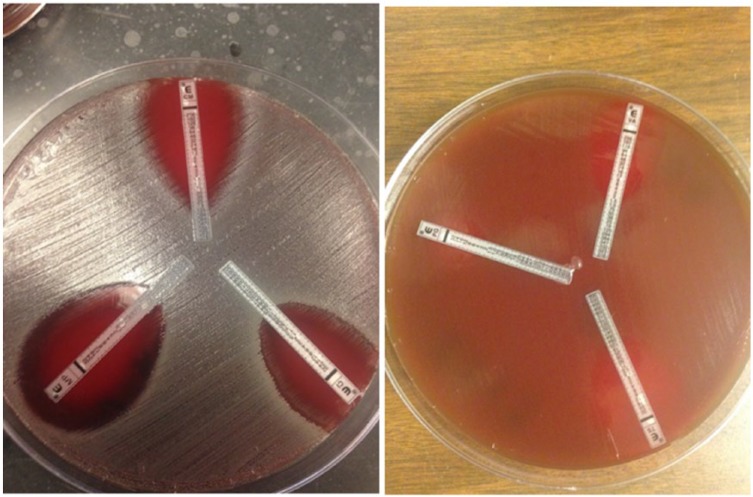
E-test of select antibiotics tested against *Clostridium tertium*. E-testing was performed to evaluate the susceptibility of *C tertium* to select antibiotics. The organism was susceptible to vancomycin, ciprofloxacin, sulfamethoxazole/trimethoprim, meropenem, and clindamycin.

These case reports demonstrate that *C tertium* is a rare cause of SBP. However, *C tertium* is often mistaken for *Bacillus* or *Lactobacillus* species because of its micromorphology and growth pattern (Figure 1), which could lead to underdiagnoses.^14-16^ Therefore, thorough identification of this species is critical. Unfortunately, traditional identification of anaerobes is time-consuming and cumbersome. New methods based on mass spectrometry such as MALDI-TOF mass spectrometry, which have been developed for the rapid identification of bacterial strains, can offer new possibilities for identification.^15,16^ Our case report is unique as we identified *C tertium* by MALDI-TOF. The appropriate identification of the organism allowed for early identification and susceptibility tests to be ordered. Ultimately, the bacterial identification allowed for targeted antibiotic therapy. Targeted therapy is critical for *C tertium* because in contrast to other clostridial species, treatment can be a challenge.^5,15,16^ Although studies evaluating sufficient numbers of strains are lacking, *C tertium* exhibits resistance to various antibiotics including third- and fourth-generation cephalosporins. Additionally, our article is unique in that we performed e-testing to evaluate the susceptibility of *C tertium* to select antibiotics. The organism was susceptible to vancomycin, ciprofloxacin, sulfamethoxazole/trimethoprim, meropenem, and clindamycin (Figure 2).

*Clostridium tertium*’s sporadic presence as an infectious pathogen in SBP can make treatment decisions difficult and lead to inappropriate antibiotics. Four characteristics of *C tertium* make it a clinically important bacterium worthy of consideration in evaluating SBP patients: (1) antibiotic resistance, (2) aerotolerant nature and difficulty in identification, (3) human commensal, and (4) potential to cause mortality. *C tertium* exhibits resistance to several antibiotics including antibiotics recommended for empiric therapy for SBP.^5^ *C tertium* is often resistant to clindamycin, metronidazole, and cephalosporins; however, the *C tertium* identified in our case report was susceptible to clindamycin. For SBP patients empirically treated with guideline-recommended antibiotics, clinical monitoring is highly recommended to suspect alternative etiologic organisms.^17^ In our patient, antibiotics were selected based on expected etiology (eg, *Escherichia coli*); however, once our patient did not respond clinically, antibiotics were adjusted. Second, *C tertium* is aerotolerant, allowing the bacterium to survive the oxygen-rich environment of the ascitic fluid.^5,10^ It also provides *C tertium* the ability to grow morphologically distinct features under aerobic versus anaerobic conditions.^15,16^ When cultured under aerobic conditions, *C tertium* was initially identified as a *Lactobacillus* species in a case report, and only under careful anaerobic conditions was the proper bacterium identified.^14^ This oftentimes leads to false identification and delay in proper antibiotic treatment. The introduction of MALDI-TOF for the rapid identification of bacterial pathogens may be particularly helpful in this group of organisms. Third is *C tertium*’s presence as a normal commensal of the gastrointestinal tract. This, coupled with gastrointestinal abnormalities or immunosuppression, may set the stage for potential overgrowth and infection.^5^ Finally, *C tertium* is associated with mortality. Of the 4 case reports currently existing of *C tertium* SBP, one patient had died, two responded to therapy, and one is unknown. *C tertium* causing other types of infections has also demonstrated a mortality potential. While the majority of *C tertium* cases are associated with patients with neutropenia, there are cases demonstrating that *C tertium* is an important cause in a non-neutropenic patient.^5,8,10,13^

## Conclusion

This is a unique case of *C tertium* SBP and bacteremia in a patient with alcoholic cirrhosis. The patient was successfully treated with intravenous antibiotics and was discharged home on oral ciprofloxacin. The goal of this case report is to draw attention to the infectious potential of *C tertium*. Additionally, this case report is unique in that it is the fourth documented *C tertium* SBP case, utilized MALDI-TOF for identification, and susceptibility testing for select antibiotics was performed.

## References

[1] Wiest R Krag A Gerbes A Spontaneous bacterial peritonitis: recent guidelines and beyond. Gut. 2012;61:297-310. 22147550 10.1136/gutjnl-2011-300779

[2] Singh N Wagener MM Gayowski T Changing epidemiology and predictors of mortality in patients with spontaneous bacterial peritonitis at a liver transplant unit. Clin Microbiol Infect. 2003;9:531-537. 12848729 10.1046/j.1469-0691.2003.00691.x

[3] González Alonso R González García M Albillos Martínez A Physiopathology of bacterial translocation and spontaneous bacterial peritonitis in cirrhosis [in Spanish]. Gastroenterol Hepatol. 2007;30(2):78-84. 17335715 10.1157/13099277

[4] Barshak M Kasper DL Intraabdominal infections and abscesses. In: Kasper D Fauci A Hauser S Longo D Jameson J Loscalzo J, eds. Harrison’s Principles of Internal Medicine. 19th ed. New York, NY: McGraw-Hill; 2015.

[5] Miller DL Brazer S Murdoch D Reller LB Corey GR Significance of *Clostridium tertium* bacteremia in neutropenic and nonneutropenic patients: review of 32 cases. Clin Infect Dis. 2001;32:975-978. 11247721 10.1086/319346

[6] Salvador F Porte L Durán L et al Breakthrough bacteremia due to *Clostridium tertium* in a patient with neutropenic fever, and identification by MALDI-TOF mass spectrometry. Int J Infect Dis. 2013;17:e1062-e1063. 23823278 10.1016/j.ijid.2013.03.005

[7] Vanderhofstadt M André M Lonchay C et al *Clostridium tertium* bacteremia: contamination or true pathogen? A report of two cases and a review of the literature. Int J Infect Dis. 2010;14(Suppl 3):e335-e337. 20598605 10.1016/j.ijid.2010.02.002 PMC7129576

[8] Butler T Pitt S Spontaneous bacterial peritonitis due to *Clostridium tertium*. Gastroenterology. 1982;82:133-134. 7053324

[9] King BM Ranck BA Daugherty FD Rau CA *Clostridium tertium* septicemia. N Engl J Med. 1963;269:467-469. 14032818 10.1056/NEJM196308292690909

[10] Victor GH Opal SM Spontaneous bacterial peritonitis: analysis of treatment and outcome. Can J Infect Dis. 1991;2(4):147-154. 22529726 10.1155/1991/327589 PMC3328012

[11] Ingram CW Cooper JN Clostridial bloodstream infections. South Med J. 1989;82:29-31. 2911760 10.1097/00007611-198901000-00009

[12] Ray P Das A Singh K Bhansali A Yadav TD *Clostridium tertium* in necrotizing fasciitis and gangrene. Emerg Infect Dis. 2003;9:1347-1348. 14626222 10.3201/eid0910.030287 PMC3033068

[13] Chalhoub V Kallab R El Hajj A Hachem K Yazbeck P Septic shock due to *Clostridium tertium* in an immunocompetent patient following colitis without inflammatory bowel disease. Anaesth Crit Care Pain Med. 2016;35:167-168. 26862069 10.1016/j.accpm.2015.10.007

[14] You MJ Shin GW Lee CS *Clostridium tertium* bacteremia in a patient with glyphosate ingestion. Am J Case Rep. 2015;16:4-7. 25577783 10.12659/AJCR.891287 PMC4289481

[15] Grosse-Herrenthey A Maier T Gessler F et al Challenging the problem of clostridial identification with matrix-assisted laser desorption and ionization-time-of-flight mass spectrometry (MALDI-TOF MS). Anaerobe. 2008;14:242-249. 18621134 10.1016/j.anaerobe.2008.06.002

[16] Nagy E Becker S Kostrzewa M Barta N Urbán E The value of MALDI-TOF MS for the identification of clinically relevant anaerobic bacteria in routine laboratories. J Med Microbiol. 2012;61:1393-1400. 22700545 10.1099/jmm.0.043927-0

[17] Runyon BA Management of adult patients with ascites due to cirrhosis: update 2p12. http://www.aasld.org/sites/default/files/guideline_documents/141020_Guideline_Ascites_4UFb_2015.pdf. Accessed July 10, 2017.

